# The Association of Periodontitis and Peripheral Arterial Occlusive Disease—A Systematic Review

**DOI:** 10.3390/ijms20122936

**Published:** 2019-06-15

**Authors:** Mark Kaschwich, Christian-Alexander Behrendt, Guido Heydecke, Andreas Bayer, Eike Sebastian Debus, Udo Seedorf, Ghazal Aarabi

**Affiliations:** 1 Department of Vascular Medicine, University Heart Center Hamburg, University Medical Center Hamburg-Eppendorf, 20251 Hamburg, Germany; ch.behrendt@uke.de (C.-A.B.); s.debus@uke.de (E.S.D.); 2 Department of Surgery, University Medical Centre Schleswig-Holstein, Campus Luebeck, Ratzeburger Allee 160, 23538 Luebeck, Germany; andreas.bayer@uksh.de; 3 Department of Prosthetic Dentistry, Center for Dental and Oral Medicine, University Medical Center Hamburg-Eppendorf, 20246 Hamburg, Germany; g.heydecke@uke.de (G.H.); u.seedorf@uke.de (U.S.); g.aarabi@uke.de (G.A.)

**Keywords:** oral disease, periodontitis, PD, peripheral arterial occlusive disease, PAOD, systematic review

## Abstract

**Background**: Observational studies support an association between periodontitis (PD) and atherosclerotic vascular disease, but little is known specifically about peripheral arterial occlusive disease (PAOD). Objectives: To systematically review the evidence for an association between PD and PAOD. Data Sources: Medline via PubMed. Review Methods: We searched the Pubmed database for original studies, case reports, case series, meta-analyses and systematic reviews that assessed whether there is an association between PD (all degrees of severity) and PAOD (all degrees of severity). The reporting of this systematic review was in accordance with the Preferred Reporting Items for Systematic Reviews and Meta-Analyses (PRISMA) statement following the Population, Intervention, Control, and Outcome (PICO) format. Results: 17 out of 755 detected studies were included in the qualitative synthesis. Nine studies demonstrated associations between PD and PAOD, and two studies reported associations between tooth loss and PAOD. Six studies addressed the pathomechanism regarding PD as a possible trigger for PAOD. No study that dismissed an association could be detected. Odds ratios or hazard ratios ranged from 1.3 to 3.9 in four large cohort studies after adjusting for established cardiovascular risk factors. Conclusions: The presented evidence supports a link between PD and PAOD. Further studies which address the temporality of PD and PAOD and randomized controlled intervention trials examining the causal impact of PD on PAOD are needed. Although our results cannot confirm a causal role of PD in the development of PAOD, it is likely that PD is associated with PAOD and plays a contributing role.

## 1. Introduction

As a consequence of demographic changes and the proliferation of the western lifestyle, peripheral arterial occlusive disease (PAOD) has developed into a widespread disease with globally over 200 million persons affected [1,2,3]. PAOD preferably effects the peripheral limb vessels of the lower extremity. It limits a person’s ability to walk, may require revascularization, or worse yet, can result in the loss of a limb. But it is not simply a disease of the legs. PAOD is one of multiple clinical manifestations of atherosclerotic vascular disease. It is a clinical manifestation of a systemic disease, that is frequently associated with ischemic heart disease, stroke, abdominal aortic aneurysms, and other serious health issues [4]. In addition to its clinical aspects that limit the patient’s quality of life, it has a profound cost impact on the healthcare system [5].

Despite the benefit of modern medicine, to date, there is no curative treatment available, and all non-invasive or invasive approaches aim to address the symptoms or disease progression. Hence, screening and prevention programs have gained more attention to identify new PAOD risk factors. It is well known that PAOD is associated with tobacco use, diabetes, high cholesterol, and higher age [3]. With respect to prevention of PAOD, the identification of additional risk factors is of high clinical importance.

Nowadays, there is scientific consensus that the development of PAOD is also associated with chronic subclinical inflammation [6,7,8]. The inflammatory genesis has been demonstrated to be multifactorial, and there is evidence that local inflammatory processes can spread systemically and trigger inflammation of the vessel wall [9]. One inflammatory focus in humans is the oral cavity, and it was proposed that chronic oral inflammations, e.g., periodontitis (PD) and caries, lead to degradation of the tooth-supporting structures [8] and may, in concert with other established risk factors, be able to trigger PAOD. PD is caused by the outgrowth of oral microorganisms, which induce a destructive host inflammatory response that contributes to progressive periodontal tissue destruction and loss of the alveolar bone around the teeth, resulting in gingival pocket formation and clinical attachment loss and, if untreated, tooth loss [10]. Tailored options are available for periodontitis treatment at various stages [11]. Approximately 47% of adults aged ≥30 years in the United States have chronic periodontitis (CP), with 30% having moderate and 8.5% severe PD [12]. PD is a complex inflammatory disease, with genetic and epigenetic factors having a role along with lifestyle and environmental factors, such as smoking, oral hygiene, nutrition, and stress, as well as other widespread systemic diseases, such as diabetes. Hereditary factors, age-dependent mutagenesis, and epigenetic changes have also been involved in the development of certain head and neck cancers, such as squamous cell carcinomas [13]. Chronic PD and obesity have been shown to be associated with pro-atherogenic lipid profiles, which are also risk factors for PAOD [14].

PD has been shown to be associated with atherosclerotic vascular disease. At least four basic pathogenic mechanisms are currently hypothesized that may explain how PD may promote atherosclerosis [15]: (1) Oral bacteria enter the blood stream and invade the arterial wall by chronic low level bacteremia; (2) inflammatory mediators released from the sites of the oral inflammation into the blood stream cause an acute phase reaction, which is pro-atherogenic; (3) specific components of oral pathogens trigger a host immune response, thereby promoting autoimmunity; (4) specific bacterial toxins that are produced by oral pathogenic bacteria have pro-atherogenic effects. A connection between PD as a trigger for the development of PAOD has been controversially discussed for years.

Therefore, the goals of this systematic review were: (1) to collect evidence for an association between PD or tooth loss and PAOD from the published literature and (2) to look for clinical studies that investigated the pathomechanism that might be involved in a potential cause-effect relationship between PD and PAOD.

## 2. Results

**Figure 1:**
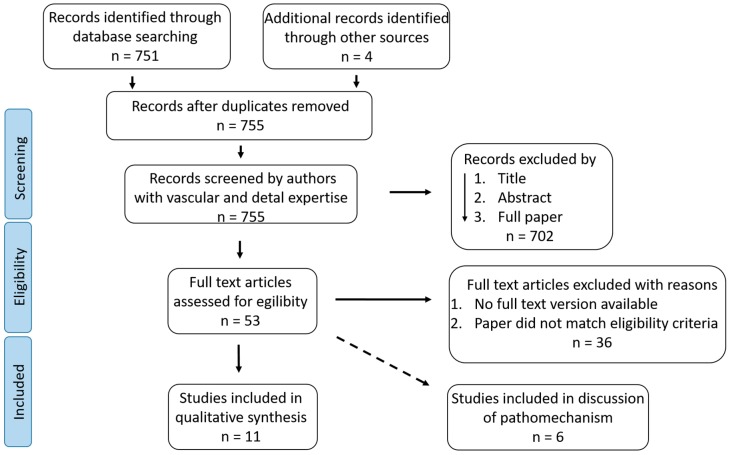
Preferred Reporting Items for Systematic Reviews and Meta-Analyses (PRISMA) flow diagram, the detected studies regarding the pathomechanism were also inserted into the PRISMA diagram (represented by a dashed line).

Seven hundred and fifty-one studies were detected by the search formula (shown in the method section), and four additional studies were identified through other sources and added in December 2018. Seven hundred and two studies were excluded following the screening algorithm (see Methods section). Full-text articles were assessed for eligibility. Thirty-six studies were excluded because no full-text article was available, the paper did not match the eligibility criteria, or it had limitations regarding the definition of PAOD or PD. Following application of the eligibility criteria, 17 studies out of the detected 755 studies were included in the qualitative synthesis. Nine studies demonstrated associations between PD and PAOD and two studies associations between tooth loss and PAOD. Six studies addressed the pathomechanism regarding PD as a trigger for PAOD and were included in the discussion. No study that dismissed an association was detected. Figure 1 summarizes the search-results using the Preferred Reporting Items for Systematic Reviews and Meta-Analyses (PRISMA) flow diagram.

### 2.1. Association between PD or Tooth Loss and PAOD

The first study to demonstrate an association between PD and PAOD was a longitudinal study published by Mendez et al. in 1998 [16]. One thousand, one hundred and ten men were followed for up to 30 years, and a 2.27-fold increased incidence rate (95% CI: 1.32–3.9, *p* = 0.003) of developing PAOD was observed in men with clinically significant PD at baseline compared to men with no or only mild PD at baseline. Subsequently, an evaluation of the prospective Health Professionals Follow-up Study [17], which was based on 45,136 male health professionals free of cardiovascular diseases at baseline and 342 incidences of PAOD that had occurred during 12 years of follow-up, showed that incident tooth loss caused by PD was significantly associated with elevated risk of PAOD (relative risk (RR) for history of PD: 1.41, RR for any tooth loss during follow-up: 1.39, after controlling for traditional risk factors of cardiovascular disease) [17].

In a cross-sectional evaluation of data from the National Health and Nutrition Examination Survey (NHANES), 172 of 3585 participants were diagnosed to have PAOD. PD was significantly associated with PAOD with an odds ratio (OR) of over two in men and women [18]. Moreover, systemic markers of inflammation, such as C-reactive protein (CRP), white blood cell count, and fibrinogen, were also associated with PAOD and PD.

Munoz-Torres et al. [19] assessed the association between baseline number of teeth and recent tooth loss and the risk of PAOD in over 70,000 women participating in the Nurses’ Health Study. During 16 years of follow-up, a significant association between incident tooth loss and the hazard of PAOD (hazard ratio (HR) = 1.31 95% CI: 1.00–1.71) could be demonstrated.

A recently reported meta-analysis that included a total of 4,307 participants from seven independent studies, confirmed these findings. The study showed a significantly increased risk of PD in PAOD patients compared with non-PAOD patients (RR = 1.70, 95% CI = 1.25–2.29, *p* = 0.01). PAOD patients also had more missing teeth than non-PAOD participants (weighted mean difference, WMD = 3.75, 95% CI = 1.31–6.19, *p* = 0.003), while no significant difference was found with respect to the clinical attachment loss between PAOD patients and non-PAOD participants (WMD = −0.05, 95% CI = −0.03–0.19, *p* = 0.686) [20].

Association between PD and PAOD was not only detected in cross-sectional and longitudinal studies, but also in several case-control studies. A strong association was observed by Soto-Barreras et al. [21] based on a small case-control study that included 30 patients with PAOD and 30 healthy controls. Patients with ≥30% of the teeth with an attachment loss ≥4 mm had a six-fold increased risk of PAOD compared to controls (OR = 8.18, 95% CI = 1.21 to 35.23, *p* = 0.031). The results also indicated that PAOD patients had higher CRP levels (*p* = 0.0413) and a higher mean decayed missing filled teeth (DMFT) index value (*p* = 0.0002) along with an elevated number of missing teeth (*p* = 0.0459) compared to the control group. The study also addressed the potential mechanism of the association. The CRP level was significantly higher (*p* = 0.0413), and there was also a difference in the decayed-missing-filled-teeth (DMFT) index (*p* = 0.0002), with a higher number of missing teeth (*p* = 0.0459) in the PAOD group compared to the control group. However, there were no significant differences regarding the frequency of bacteria in serum and subgingival plaque samples.

A strong association between PAOD and PD was also reported by Calapkorur et al. [22] who found an OR of 5.8 after adjusting for confounders (age, gender, diabetes, hypertension, and body mass index (BMI)) based on a case-control study including 40 patients with PAOD and 20 healthy controls. In a multicenter, population-based, case-control study that included 212 young women with PAOD and 475 healthy women from the Netherlands, PD was associated with PAOD with an OR of 3.0 (95% CI: 1.4–6.3) [23].

In a case-control, retrospective study based on chart reviews, Molloy et al. [24] evaluated self-reported systemic conditions and smoking history of 2006 selected patients attending the University of Minnesota dental clinics. In addition, the number of missing teeth and the degree of alveolar bone loss were recorded. After adjustments for age, sex, diabetes, and smoking, vascular disease and vascular surgery were significantly associated with alveolar bone loss and the number of missing teeth. The association could be demonstrated not only in people of mostly European descent but also in Asians. Ahn et al. observed an OR of 2.03 (95% CI: 1.05–3.93) for the association between severe PD and PAOD in a Korean community cohort of adults aged over 40 years (N = 1343) [25].

Chen et al. [26], observed that PD was significantly associated with PAOD (OR: 5.45, 95% CI: 1.57−18.89 after adjusting for age, gender, diabetes, and smoking) in a Japanese case-control sample of 25 patients with aorto-iliac and/or femoro-popliteal occlusive disease and 32 generally healthy patients who were employed as controls. Table 1 summarizes the results presented above.

### 2.2. Pathomechanism

**Figure 2:**
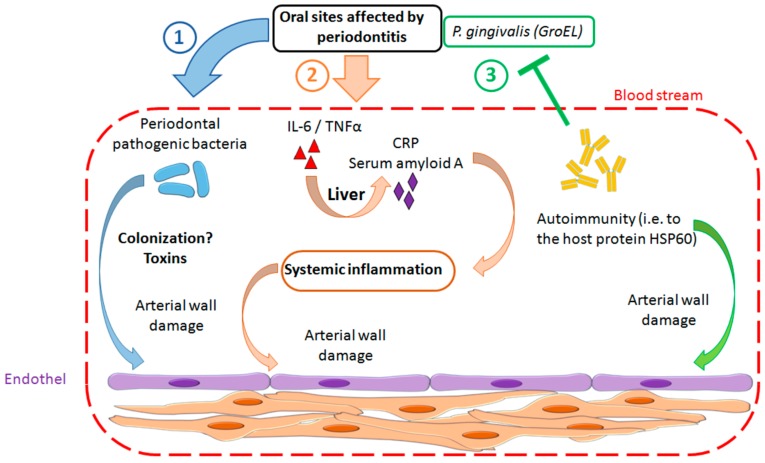
Potential pathomechanism that may be involved in the association of PD and PAOD; (1) periodontal pathogenic bacteria enter the bloodstream, invade and damage the arterial wall; (2) release of inflammatory mediators into the bloodstream, such as serum amyloid A, interleukin-6 (IL-6), tumor necrosis factor α (TNFα), and C-reactive protein (CRP) causing a systemic inflammation that also damages the arterial wall; (3) autoimmunity to host proteins (i.e., heat shock protein 60, HSP60) resulting from the host immuno response to the bacterial proteins, such as the HSP60 homolog GroEL.

For this systematic review, six studies were found that addressed the potential pathomechanism that may be involved in the association and may explain how PD could induce or aggravate PAOD (see supplementary Table S1 for details). These studies suggest at least three basic mechanisms (Figure 2): (1) Periodontal pathogenic bacteria were demonstrated to enter the bloodstream and to invade atherosclerotic lesions at damaged sites of the arterial wall [27,28]; (2) experimental data showed that inflammatory mediators, such as serum amyloid A and anti-inflammatory mediators, are released from the oral sites affected by PD into the bloodstream, thereby modulating systemic inflammation [29,30]; (3) it was demonstrated in patients with PD that autoimmunity to the host protein heat shock protein 60 (HSP60) resulted from the host immune response to the bacterial HSP60 homolog GroEL produced by *Phorphyromonas gingivalis* (the main oral pathogens involved in PD) [31].

In a blinded randomized controlled trial, Li et al. [32] could show that treatment of PD lowered the number of circulating CD34+ cells relative to untreated controls. The reduction of circulating CD34+ cells correlated with the treatment-induced decrease in sites showing bleeding on probing and the number of periodontal pockets with a depth of ≥4 mm, suggesting that treatment of PD reduced the level of systemic inflammation. On the other hand, treatment of PD did not improve endothelial function in this study.

## 3. Discussion

This systematic review supports that PD is associated with PAOD, which may lead to the hypothesis that PD may be a risk factor for PAOD. To date, many studies describe associations between periodontitis or oral disease and atherosclerosis in general. Therefore, we wanted to specifically focus on studies that concern peripheral vascular disease (PAOD) as a potential consequence of periodontitis in this systematic review. However, it has to be stated that the published literature is not absolutely certain about the term PAOD for “peripheral artery occlusive disease”. In publications, it is frequently used for lower extremity artery disease (LEAD). Indeed, other peripheral localizations, including the carotid and vertebral, upper extremities, mesenteric and renal arteries, are also frequently affected, mainly by atherosclerosis, and complete the family of peripheral arterial diseases [6]. In addition, there is sometimes no differentiation between extracranial and cerebral atherosclerotic pathologies. Hence, for this systematic review, we excluded studies that were linked to carotid/cerebral sclerosis, coronary sclerosis as well as vascular sclerosis in general. We also excluded animal studies as we wanted to focus on clinical evidence for an association between the two pathologies.

All studies that were included in this systematic review could detect an association between PD or tooth loss and PAOD irrespective of study design, outcome measure, and study population.

This supports that the consistency of the association is high. It must be noted, however, that there is inherent bias, since risk factors for PAOD also can cause PD. Standardized effect sizes, which provide a measure of the strength of the association, have mostly been determined by logistic regression analyses and reported as ORs together with 95% CIs. After making adjustments for age, gender, and other cardio vascular disease (CVD) risk factors, the reported ORs ranged from somewhat over two in NHANES [18] to over eight in the small case-control study published by Soto-Barreras et al. [21]. In general, the smaller case-control studies yielded higher ORs than the larger cohort studies. Measures of hazard ratios or relative risk estimates are available from only a few studies. Data from the Nurses’ Health Study demonstrated a significant association between incident tooth loss and PAOD and reported a hazard ratio of 1.3 for PAOD in women with PD vs. women without PD. The meta-analysis published by Yang et al. 2018 [20] reported a statistically significant relative risk of 1.7 for PAOD in people with PD vs. those without PD.

Taken together, these results suggest that severe PD increases the risk for PAOD to a similar extent as PD increases the risk for cardiovascular events, which with respect to the latter was shown to be ≈1.20-fold in adjusted models from meta-analyses of prospective cohort studies [33,34]. Smoking, a profound risk factor for PAOD, is associated with PAOD with odds ratios ranging between 1.7 and 7.4 [3]. With respect to diabetes, a twofold increased rate of macroalbuminuria and a threefold increased rate of end-stage renal disease were found in diabetics who also had severe periodontitis compared to diabetics without severe periodontitis [35]. Moreover, cardiorenal mortality resulting from ischaemic heart disease and diabetic nephropathy was three times higher in diabetics with severe PD compared to periodontally healthy diabetics [36]. The risk of PD for preterm delivery ranged between 4.45 and 7.07, depending on the gestational age [37]. Severe maternal PD was also shown to be associated with preterm low birth weight with an odds ratio of 7.5 [38]. All referenced studies considered a wide range of suspected confounders and included corresponding adjustments. Thus, PD may be a risk factor for multiple, widespread diseases. On the other hand, the possibility that some of the weak associations may be due to residual confounding by unrecognized confounders should not be neglected.

If PD is a causal or, at the least, an important contributor involved in the pathogenesis of PAOD, one would expect that PD precedes the onset of PAOD. However, only very limited information exists with respect to the temporality of both diseases. According to results from the prospective Health Professionals Follow-up Study, tooth loss seemed to precede PAOD, since the incidence of PAOD was most strongly associated with tooth loss in a period of 2 to 6 years prior to the occurrence of PAOD [17]. The fact that tooth loss in the previous 2 to 6 years was more strongly associated with PAOD than tooth loss in the previous 2 years or 6 to 8 years suggests that 6 years may be too distant and 2 years may be too recent for tooth loss to have an impact on PAOD. However, these reported time-dependent differences in the strength of the association were based on only the disparity of only a few PAOD incidences and may, thus, have been chance findings.

The plausibility of a causal or, at least, an important involvement of PD in the development of PAOD mostly relates to experimental data showing that inflammation is involved in the pathophysiology of atherosclerosis, which in turn is involved in the development of PAOD. This inflammation could be caused by a direct involvement of periodontal pathogenic bacteria, which enter the vascular wall via the bloodstream. The study by Figuero et al. used nested polymerase chain reaction (PCR) to detect three periodontal pathogens in subgingival, vascular, and blood samples. Although positive test results were obtained in high fractions of the subgingival samples (>70%) and the vascular and blood samples (7 to 11.4%), patients with and without PD did not differ with respect to the levels of the targeted bacteria. Therefore, a direct involvement of the bacteria seems inconclusive at this stage.

The studies by Nishida et al. 2016 [30] and Armingohar et al. 2015 [29] support that inflammatory mediators, such as serum amyloid A and anti-inflammatory mediators, such as interleukin-10, which are released from the oral sites affected by PD into the bloodstream, thereby modulating systemic inflammation may be involved in the pathomechanism of PD-induced PAOD. In addition, autoimmunity induced by PD via the immune response of the host to the bacterial HSP60 homolog GroEL produced by *P. gingivalis* (the main oral pathogen involved in PD) could play a role [31]. Support for this mechanism comes from the study by Choi et al., who successfully established *P. gingivalis*–specific T-cell lines from atheroma lesions isolated from PD patients. However, the study included only two patients, and the origin of the lesions remained unclear.

**Figure 3:**
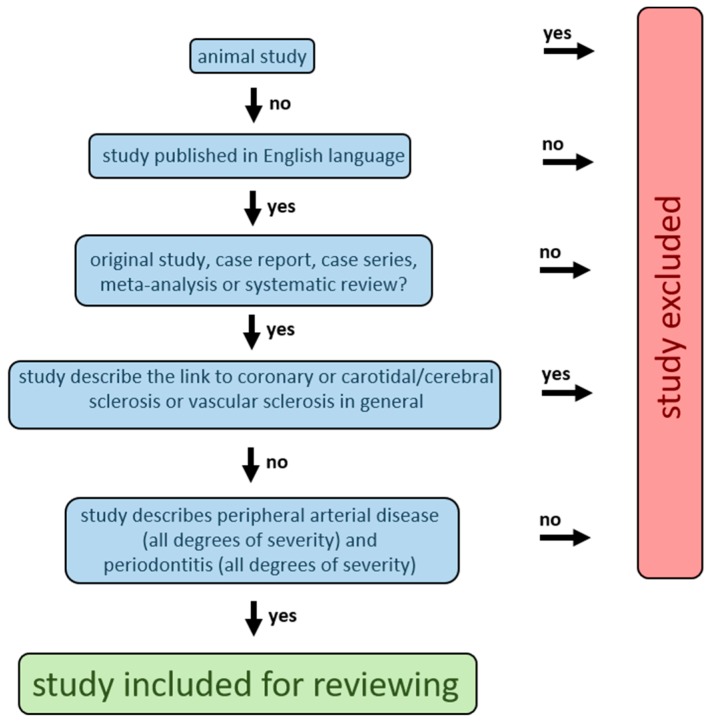
Screening algorithm.

It is evident from the results section that our search-strategy yielded only a few publications that dealt with the pathomechanism of the association between PD and PAOD. A large fraction of the published mechanistic studies concerned animal, in vitro and ex vivo studies describing the link between PD and vascular sclerosis in general rather than that between PD and PAOD specifically. These studies were, however, not eligible for this review based on the pre-defined exclusion criteria shown in Figure 3. Roles of oral infections in the pathomechanism of atherosclerosis, in general, were discussed in great detail in a recent review published by Aarabi et al. [15]. Briefly, there is a wealth of support for at least four plausible pathogenic mechanisms: (1) low-level bacteremia by which oral bacteria enter the bloodstream and invade and damage the arterial wall; (2) systemic inflammation induced by inflammatory mediators, which are released from the sites of the oral inflammation into the bloodstream; (3) autoimmunity to host proteins which results from the host immune response to specific components of oral pathogens; (4) pro-atherogenic effects resulting from specific bacterial toxins that are produced by oral pathogenic bacteria. In addition, recent genome-wide association studies supported that PD and PAOD share at least one important predisposing genetic risk haplotype that is located at chromosome 9p21.3 in a locus known as *ANRIL*/*CDKN2B-AS1* [39,40]. The risk haplotype affects the structure and expression of ANRIL, which is a long non-coding RNA (lncRNA) that, such as other lncRNAs, regulates genome methylation, thereby affecting the expression of multiple genes by *cis* and *trans* mechanisms. How precisely ANRIL contributes to the risk of PD and PAOD on the molecular level is currently unclear.

So far, many, but not all, studies demonstrated the presence of bacterial DNA in a large number of atheromas, but only very few could demonstrate the successful isolation of viable bacteria from an atherosclerotic plaque. In fact, to the best of our knowledge, there is not a single study available that could demonstrate isolation and cultivation of viable *P. gingivalis* from atherosclerotic tissue. In addition, it should be noted that long-term treatment with antibiotics, such as roxithromycin and rifalazil, showed no benefit in patients with an established diagnosis of PAOD [41,42]. Nevertheless, it seems prudent at this stage to recommend that patients with PAOD should be routinely referred to a dentist, and periodontitis should be appropriately treated if present.

## 4. Methods

### 4.1. Literature Search

This systematic review considered all studies listed in PubMed until 30 September 2018. For additional studies, we double-checked in EMBASE and supplemented with additional hits obtained from Google Scholar. Grey literature was not part of the review process. It was reported in accordance with the Preferred Reporting Items for Systematic Reviews and Meta-Analyses statement (PRISMA) [43]. For the PRISMA checklist, see http://www.prisma-statement.org. We employed the Population, Intervention, Control, and Outcome (PICO) format to answer the following PICO questions:

Is PD associated with the occurrence of PAOD?

Population = All patients with PD (all degrees of severity) who were detected in the selected literature

Intervention = None

Comparison = Patients with and without PD

Outcome = PAOD of the lower extremity

We first determined a list of synonyms and MeSH-terms (Medical Subject Headings) for PAOD and PD. Using these lists, we defined the following search-formula.

(peripheral arterial disease OR peripheral artery disease OR PAD OR occlusive vascular disease OR IC OR intermittent claudication OR CLI OR peripheral arterial occlusive disease OR peripheral artery occlusive disease OR PAOD OR lower limb ischemia OR DFS OR vascular surgery)

AND (periodontitis OR gum disease OR pyorrhea OR periodontal disease OR periodontal infection OR periodontal conditions OR chronic periodontitis OR periodontal health OR tooth loss OR attachment loss OR probing pocket depth)

### 4.2. Study Selection and Data Extraction

**Figure A1:**
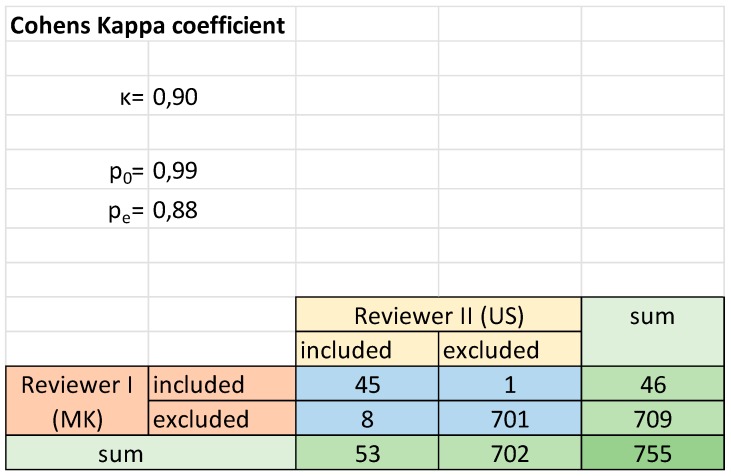
Cohens Kappa coefficient calculation.

All studies were reviewed by two independent authors, one with vascular expertise (MK) and one with oral health expertise (US). Reviewers were blinded to each other results. Both reviewers screened all papers selected by the search formula to identify inclusion criteria. Any disagreements between reviewers at each stage of selection were resolved by consensus. Cohen’s kappa coefficient (*κ*) demonstrated good agreement between the two reviewers (*κ* = 0.9) (Appendix A Figure A1). The studies were processed according to the PRISMA flow diagram shown in Figure 1. To sort the studies, we developed a screening-algorithm (Figure 3).

Following this algorithm, we decided whether the eligibility criteria were met; first by title, and if the title led to an uncertainty regarding the eligibility criteria, additionally by the abstract. If there was still uncertainty, the whole paper was read. Animal studies, studies that were published in non-English, studies that did not correspond to original papers, case reports, case series, meta-analyses, or systemic reviews were excluded. In addition, studies on coronary or carotidal/cerebral sclerosis or vascular sclerosis were excluded in general because the aim of this systematic review was to focus on PAOD. Studies describing an association between PD (all degrees of severity) and PAOD (all degrees of severity) were included and further processed according to the PRISMA flow diagram (Figure 1). Studies that were identified by additional manual searches were also added. No duplicates were identified. Citations were managed throughout the different stages of preparing the review with the Mendeley reference management software.

### 4.3. Quality Assessment

**Figure 4:**
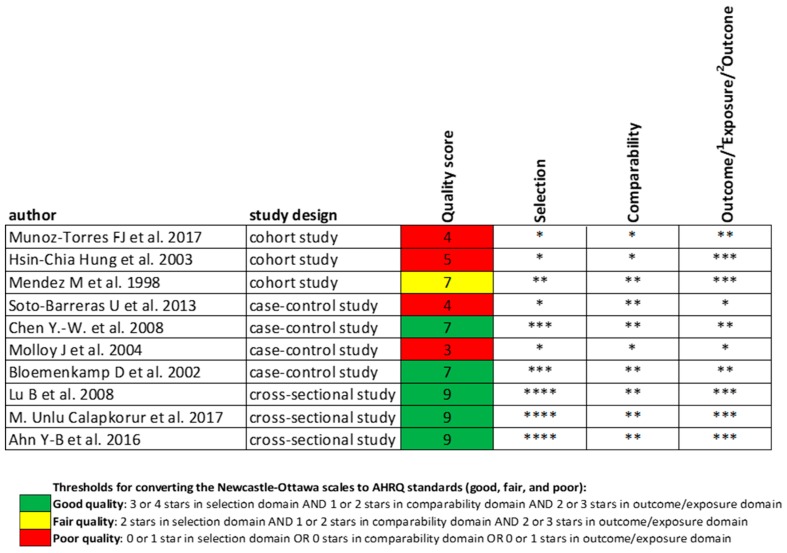
Results of the quality assessment.

The risk of bias was assessed by using the Newcastle–Ottawa Scale [44] for case-control studies and cohort studies. For the quality assessment of cross-sectional studies, we used a modified version of the Newcastle–Ottawa Scale [45]. The results of the quality assessment are shown in Figure 4.

## 5. Conclusions

In conclusion, the present evidence supports a link between PD and PAOD. Further studies which address the temporality of PD and PAOD are warranted. Thus, a causal, or at least, an important contributing role of PD in the development of PAOD can currently not be confirmed but may be suspected. Clearly, the ultimate proof of causality would depend on data from randomized controlled invention trials to show that treatment of PD can diminish or even prevent PAOD. Such data does, to the best of our knowledge, currently not exist.

## Figures and Tables

**Table 1:** Summary of the strength of the association between PD or tooth loss and PAOD. RR = risk ratio; OR = odds ratio; HR = hazard ratio; WMD = weighted mean difference. * PAOD patients had more missing teeth than non-PAOD participants. ** conditions that were significantly related to bone loss or number of missing teeth.

Ref	Study Design	Strength of the Association between PD or Tooth Lost and PAOD	Participants	Limitations
[20]	systematic review and meta-analysis	RR = 1.70 (95% CI: 1.3–2.3; *p* = 0.01) * WMD = 3.75 (95% CI: 1.3–6.2; *p* = 0.003)	4.307	
[22]	cross-sectional study	OR = 5.8 (95% CI: 1.5–21.9; *p* = 0.009)	60	
[19]	cohort study	HR = 1.3 (95% CI: 1.0–1.7)	79.663	no adjustment for smoking
				only women
[25]	cross-sectional study	OR = 2.0 (95% CI: 1.0–3.9; *p* = 0.036)	1.343	
[21]	case-control study	OR = 8.2 (95% CI: 1.2–35.2; *p* = 0.031)	60	
[26]	case-control study	OR = 5.5 (95% CI: 1.6–18.9; *p* = 0.007)	57	
[18]	cross-sectional study	OR = 2.3 (95% CI: 1.2–4.2; *p* = 0.004)	3.585	
[24]	case-control study	**vascular disease *p*-value 0.014; **vascular surgery *p*-value 0.001	2.006	
[17]	cohort study	RR = 1.41 (95% CI: 1.1–1.8)	45.136	only men
[23]	case-control study	OR = 3.0 (95% CI: 1.4–6.3)	687	only women
[16]	cohort study	OR = 2.27 (95% CI: 1.3–3.9; *p* = 0.003)	1.110	only men

## Supplementary Materials

Supplementary Materials can be found at https://www.mdpi.com/1422-0067/20/12/2936/s1.
